# Buffalo Dispensary Appointments for 1864

**Published:** 1863-12

**Authors:** 


### BUFFALO DISPENSARY APPOINTMENTS FOR 1864.

| | | |
|---|---|---|
| Dr. J. N. Brown, | Physician | 1st Ward. |
| " P. H. Strong, | " | 2d " |
| " J. Boardman, | " | 3d " |
| " J. R. Lathrop, | " | 4th " |
| " J. Hauenstein, | " | 5th " |
| " J. B. Samo, | " | 6th " |
| " S. Eastman, | " | 8th " |
| " C. C. Wyckoff, | " | 9th " |
| " J. Whittaker, | " | 10th " |
| " L. P. Dayton, | " | 11th " |
| " Henry Nichell, | " | 12th " |

Dr. T. F. Rochester, and<br>" G. N. Burwell, } *Consulting Physicians.*

Dr. J. P. White, and<br>" J. F. Miner, } *Consulting Surgeons.*

W. H. Peabody, *Apothecary.*

S. N. Callender, *Treasurer.* Jason Sexton, *President.*

Isaac D. White, *Secretary.* F. P. Wood, *Vice President.*

